# BTB-BACK-TAZ domain protein MdBT2-mediated MdMYB73 ubiquitination negatively regulates malate accumulation and vacuolar acidification in apple

**DOI:** 10.1038/s41438-020-00384-z

**Published:** 2020-09-02

**Authors:** Quan-Yan Zhang, Kai-Di Gu, Jia-Hui Wang, Jian-Qiang Yu, Xiao-Fei Wang, Shuai Zhang, Chun-Xiang You, Da-Gang Hu, Yu-Jin Hao

**Affiliations:** 1 National Key Laboratory of Crop Biology, Shandong Collaborative Innovation Center of Fruit & Vegetable Quality and Efficient Production, College of Horticulture Science and Engineering, Shandong Agricultural University, Tai-An, Shandong 271018 China; 2 College of Chemistry and Material Science, Shandong Agricultural University, Tai-An, Shandong 271018 China

**Keywords:** Translation, Plant molecular biology

## Abstract

As an important primary metabolite, malate plays a key role in regulating osmotic pressure, pH homeostasis, stress tolerance, and fruit quality of apple. The R2R3-MYB transcription factor (TF) MdMYB73 was identified as a protein that plays a critical role in determining malate accumulation and vacuolar acidification by directly regulating the transcription of aluminum-activated malate transporter 9 (*MdALMT9*), vacuolar ATPase subunit A (*MdVHA-A*), and vacuolar pyrophosphatase 1 (*MdVHP1*) in apple. In addition, the bHLH TF MdCIbHLH1 interacts with MdMYB73 and enhances the transcriptional activity of *MdMYB73*. Our previous studies demonstrated that the BTB-BACK-TAZ domain protein MdBT2 can degrade MdCIbHLH1 to influence malate accumulation and vacuolar acidification. However, the potential upstream regulators of MdMYB73 are currently unknown. In this study, we found that MdBT2 directly interacts with and degrades MdMYB73 through the ubiquitin/26S proteasome pathway to regulate malate accumulation and vacuolar acidification. A series of functional assays with apple calli and fruit showed that MdBT2 controls malate accumulation and vacuolar acidification in an MdMYB73-dependent manner. Overall, our findings shed light on the mechanism by which the BTB-BACK-TAZ domain protein MdBT2 regulates malate accumulation and vacuolar acidification by targeting MdMYB73 and MdCIbHLH1 for ubiquitination in apple. This information may help guide traditional breeding programs and fruit tree molecular breeding, and lead to improvements in fruit quality and stress tolerance.

## Introduction

Malate, a major intermediate in several metabolic processes, is present in most plant species and plays critical roles in regulating osmotic pressure, pH homeostasis, nutrient absorption, and stress resistance^1,2^. Malate also acts as an important acidity indicator for fruit quality that influences the perception of its sweetness, the accumulation of other nutrients, and the processing quality of fruit wine and juice^3–7^. Malate metabolism is a complex biological process and is comprised of synthesis, degradation, and transport^7,8^.

Malate is synthesized during cytoplasmic glycolysis, the mitochondrial tricarboxylic acid cycle, the chloroplast Calvin cycle, and the glycosome glyoxylate cycle^9–11^. Malate transport involves vacuolar proton pumps, and malate transporters play key roles in malate transport from the cytosol into the vacuole, thus driving vacuolar acidification^2^. Vacuolar proton pumps contain vacuolar H^+^-ATPase (V-ATPase) and H^+^-pyrophosphatase (V-PPase)^12^. V-ATPase has a complex structure that includes two functional parts: the peripheral V_1_ sector and the membrane-embedded V_0_ sector^13,14^. V_1_ has eight different subunits, with A and B subunits critical for the catalytic activity, and C–H subunits linked to hydrophobic and membrane-embedded V_0_, as is critical for ATP hydrolysis^12,15^. The V_0_ sector contains six subunits (VHA-a, -c, -c′, -c″, -d, and -e) that form a proton-conducting channel through which protons are translocated^12,16^. V-PPase, on the other hand, is composed of a single polypeptide^17,18^. V-ATPase and V-PPase are both necessary not only for forming and maintaining the electrochemical potential gradient on the tonoplast^2,19^, but also for supplying energy to other transport proteins, such as the malate transporters tDT, Ma1, and Ma10 (refs. ^7,12,20–22^).

Transcription factors (TFs) are widely involved in the regulation of malate synthesis and transport. For example, SlWRKY42 directly regulates *SlALMT9*, negatively affecting malate concentration in tomato^5^. In apple, allelic variations in the MYB TF *MdMYB44* impact malate concentration^23^. In addition, MdMYB1 controls malate accumulation and vacuolar acidification by directly regulating the transcription of *MdVHA-Bs, MdVHA-E2*, and *MdtDT*^12^. MdMYB73 is involved in malate accumulation and vacuolar acidification by directly regulating the transcription of *MdVHA-A*, *MdVHP1*, and *MdALMT9* (ref. ^2^). MdCIbHLH1, a bHLH TF, interacts with MdMYB73 and enhances its effects on targets to affect malate concentration and vacuolar acidification^2,24^. Studies focused on the transcriptional regulation of malate have been carried out in apples, but the effects of posttranslational regulation on malate accumulation and vacuolar acidification are still poorly understood.

BT2 is a BTB protein that functions in the regulation of telomerase in *Arabidopsis*^25^. As a cytoskeletal protein, BT2 interacts with the cullin scaffolding protein CUL3 and the RING finger protein RBX1 to form E3 ubiquitin ligase complexes^26,27^. Recent studies have shown that BT2 is a central component of an interconnected signaling network that detects and responds to multiple inputs, such as light, temperature, carbon and nitrogen levels, hormones, and stresses^28–31^. Recently, we found that the ubiquitination-related scaffold protein MdBT2 can regulate the stability of MdCIbHLH1 via ubiquitination in response to nitrate, which in turn transcriptionally reduces the expression of malate-associated genes, thereby controlling malate accumulation and vacuolar acidification in apple^24^. In contrast to MdCIbHLH1, MdMYB73 can directly regulate the transcription of malate-related genes to adjust malate accumulation and vacuolar acidification^2^. However, the potential regulators of MdMYB73 during malate accumulation are currently unknown.

In this work, we found that MdBT2 is a possible MdMYB73 interactor by employing a yeast two-hybrid (Y2H)-based screening library. We determined that this interaction represented another ubiquitination mechanism affecting malate accumulation, in addition to the ubiquitination of MdCIbHLH1 by MdBT2. Subsequently, we discovered that MdBT2 regulates the stability of MdMYB73 via ubiquitination, which in turn transcriptionally reduces the expression of malate-associated genes, thereby altering malate accumulation and vacuolar acidification in apple. Finally, we integrated these findings into a working model to explain how MdBT2 regulates malate accumulation and vacuolar acidification in apple.

## Materials and methods

### Plant materials and growth conditions

The apple calli used in this study were induced from young embryos of the “Orin” apple (*Malus domestica* Borkh)^32^. “Orin” apple calli were grown on Murashige–Skoog medium with 1.5 mg L^−1^ 2,4-dichlorophenoxyacetic acid (2,4-D) and 0.4 mg L^−1^ 6-benzylaminopurine (6-BA) at 25 °C in the dark^33^. These calli were subcultured three times at 15-day intervals before being used for further study^34^.

### Construction of expression vectors and genetic transformation

The MdBT2-antisense (35S::anti-MdBT2), MdBT2-ovx (35S::MdBT2-MYC), and MdMYB73-ovx (35S::MdMYB73-GFP and 35S::MdMYB73-MYC) vectors were generated using pCXSN, pCAMBIA1301, pCXCN-GFP, and pCAMBIA1301 vectors, respectively^2,35,36^. The partial 5′-UTR sequences of antisense *MdMYB73* were also inserted into a pCXCN-GFP vector to generate antisense suppression vectors (35S::anti-MdMYB73). The accession numbers of MdBT2 and MdMYB73 in the GDR data libraries are MDP0000643281 and MDP0000894463, respectively. These constructs were introduced into “Orin” apple calli by *Agrobacterium*-mediated transformation using *Agrobacterium tumefaciens* strain LBA4404 (ref. ^37^). Transgenic apple calli were selected on medium that contained 300 mg L^−1^ cephalosporin and 30 mg L^−1^ hygromycin. The expression level of *MdMYB73* was determined by quantitative RT-PCR (qRT-PCR)^38^.

### Y2H assays

Y2H assays were used to verify the interaction between MdBT2 and MdMYB73. The full-length and truncated cDNAs of *MdMYB73* (amino acids 1–242, 1–80, 1–131, 81–131, and 132–242) were inserted into pGAD424, while *MdBT2* cDNAs and truncated sequences (amino acids 1–335, 1–195, 1–128, 129–195, 129–335, and 196–335) were inserted into pGBT9. The pGAD424-MdMYB73 and pGBT9-MdBT2 plasmids were transferred into the Y2H Gold strain and plated on medium lacking Trp and Leu (-T/-L) at 28 °C for 2 days. The colonies were then transferred to medium lacking Trp, Leu, Ade, and His (-T/-L/-A/-H) and cultured for the interaction assays^24^.

### Pull-down assays

The full-length cDNAs of MdBT2 and MdMYB73 were inserted into pGEX-4T or pET-32a, and subsequently expressed in *Escherichia coli* BL21 (DE3) to generate recombinant proteins MdBT2-GST and MdMYB73-HIS, respectively. The fusion proteins of MdBT2-GST, MdMYB73-HIS, and GST were used for pull-down assays with anti-HIS and anti-GST antibodies^38^.

### Bimolecular fluorescence complementation assays

The cDNAs of MdBT2 and MdMYB73 were separately inserted into the 35 S::pSPYNE-nYFP and 35S::pSPYCE-cYFP vectors to form recombinant plasmids MdBT2 + N and MdMYB73 + C, respectively. Subsequently, these recombinant plasmids were transformed into *A. tumefaciens* LBA4404 (ref. ^38^). The transformed *Agrobacterium* suspension was infiltrated into *Nicotiana benthamiana* leaves. A YFP fluorescent signal was detected to identify interactions using a confocal laser scanning microscope (Zeiss LSM 510 Meta, Jena, Germany)^29^.

### Protein degradation assays

Proteins of three types of apple calli (35S::anti-MdBT2, wild type (WT)), and 35S::MdBT2-MYC) were extracted in degradation buffer (25 mM Tris, 5 mM DTT, 10 mM NaCl, 10 mM MgCl_2_, 4 mM PMSF, and 10 mM ATP) and coincubated with MdMYB73-HIS protein at 22 °C. Subsequently, these samples were collected after 0, 2, 4, and 6 h. The reaction of these samples was then stopped by adding 2× sodium dodecyl sulfate (SDS) loading buffer (0.06 M Tris-HCl, pH 6.8; 1% SDS; 1% 2-mercaptoethanol; 10% glycerol; and 0.025% bromophenol blue). These samples were then examined by western blotting after being subjected to SDS–polyacrylamide gel electrophoresis (PAGE) on 10% gels, and transferred to polyvinylidene fluoride (PVDF) membranes (Roche) and an anti-HIS (Beyotime) antibody^36^. For the proteasome inhibitor experiments, three types of apple calli were treated with 50 µM MG132 and then extracted. Subsequently, the extracts were coincubated with MdMYB73-HIS protein^24^.

### Protein ubiquitination assays

Protein ubiquitination experiments were performed both in vivo and in vitro. For the in vitro ubiquitination assays, a protein ubiquitination solution buffer (50 mM Tris (pH 7.5), 2 mM DTT, 50 mM MgCl_2_, 2 mM ATP, 100 ng rabbit E1, 100 ng human E2, and 1 µg ubi) was prepared as previously described^24^. The MdBT2-MYC active protein was extracted from the 35S::MdBT2-MYC apple calli using a Pierce classic protein A IP Kit (Thermo Fisher Scientific, San Jose, CA, USA). Mixtures including the buffer and MdMYB73-HIS protein were incubated with or without active MdBT2-MYC protein at 30 °C for the ubiquitination assays performed with western blotting. These samples were separated by 10% SDS–PAGE and blotted onto PVDF membranes. The gel blots were probed with anti-HIS and anti-ubi (Sigma Aldrich) antibodies, and were observed via chemiluminescence with an ECL Plus detection kit (Millipore) according to the manufacturer’s instructions^39^.

For the in vivo ubiquitination assays, 35S::MdMYB73-GFP and 35S::MdMYB73-GFP + 35S::MdBT2-MYC were treated with 50 µM MG132, and then extracted using a Pierce Classic Protein A IP Kit. The resulting extractions were examined by western blotting. The proteins were separated by 10% SDS–PAGE and electroblotted onto PVDF membranes. The gel blots were probed with anti-GFP (Beyotime) and anti-ubi antibodies, and observed via chemiluminescence with the ECL Plus detection kit (Millipore) according to the manufacturer’s instructions^39^.

### qRT-PCR assays

Total RNA was extracted using an RNA Plant Plus reagent kit (Tiangen, Beijing, China) according to the manufacturer’s instructions. cDNA was synthesized with a PrimeScript^TM^ RT reagent kit (TaKaRa, Dalian, China) following the manufacturer’s instructions^38^. For qRT-PCR assays, the reactions were carried out with SYBR Green PCR Master Mix in an iCycler iQ5 system (Bio-Rad, Hercules, CA, USA)^36^. The cycle threshold (Ct) 2^−ΔΔCt^ method was used for detecting the transcript levels of malate-related genes, with MdACTIN used as the reference gene^40^. Each sample was analyzed with three biological replicates. All primers used are shown in Supplementary Table 1.

### Transient expression assay in apple fruit

For antisense suppression, 200–300 bp sequences of the *MdMYB73* and *MdBT2* genes in antisense orientation were inserted into the TRV2 vector, and TRV1 was used as an auxiliary vector^30^. The TRV2-antisense constructs and TRV1 were subsequently transformed into *A. tumefaciens* strain LBA4404. The TRV2- and TRV1-carrying *Agrobacterium* was grown overnight until an OD value of 0.6 was reached, and then collected by centrifugation at 6000 r.p.m. The cells were resuspended in 10 mM MgCl_2_, 10 mM MES, and 200 μM acetosyringone and then maintained at room temperature for another 2–3 h in the dark. The two were mixed at a 5:1 ratio just before injection^41^. The fresh-bagged apple fruits of the “Red Delicious” cultivar were collected ~20 days before harvest from the orchard and used for transient expression assays. A blue dye was first injected into the apple to observe the apple injection pathway. Different infection solutions were subsequently injected into the fruit flesh, and then, the samples were extracted based on the approximate pathway of the diffused dye. qRT-PCR assays were used to detect the expression levels of the different vectors to ensure that transient expression was realized.

### Measurement of malate concentration, V-ATPase and V-PPase activity levels, and vacuolar pH

Samples of apple calli or apple fruit were thoroughly ground in liquid nitrogen, and then extracted with 80% (v/v) ice-cold methanol^24,42^. The extracted residue was dissolved in deionized water and filtered using a 0.45-μm membrane filter^24^. The malate concentration of the filtrate was measured using high-performance liquid chromatography (HPLC)^42^. The activity levels of V-ATPase and V-PPase were determined using extracted tonoplast membranes^19^. The release of inorganic phosphate was due to the bafilomycin A1-sensitive ATP hydrolytic activity, the ATP-dependent fluorescence quenching of the pH-sensitive fluorescent probe acridine orange was due to V-ATPase H^+^ transport activity, and the production of inorganic phosphate from pyrophosphate was due to V-PPase transport activity^2,43,44^. Vacuolar pH measurements were performed using extracted protoplasts from apple calli according to previously described methods^45^. The extracted protoplasts were incubated with the pH-sensitive fluorescent dye 2′,7′-bis(2—carboxyethyl)-5(6)-carboxyfluorescein acetoxymethyl ester (BCECF-AM; Molecular Probes, Eugene, OR) and then measured using a confocal microscope (Zeiss LSM 510 Meta). Vacuolar pH was quantified using the ratio of 488 and 458 nm excitation wavelengths via Zeiss LSM confocal software^12^.

### GUS analysis

The 1689-bp promoter fragment upstream of the ATG initiation codon of *MdALMT9* was inserted into pCAMBIA1300 and transformed into *A. tumefaciens* strain LBA4404 to obtain transgenic apple calli (proMdALMT9::GUS). GUS assays were conducted to verify the effect of MdBT2 on the transcriptional activity of *MdALMT9*. GUS assays were performed using a fluorescence spectrometer (Thermo Scientific) according to previously described methods^34^.

### Statistical analysis

At least three biological replicates were performed for each experiment. The data are presented as the means ± standard deviation unless otherwise indicated. Statistical analysis was carried out via Tukey’s single factor test using DPS software. For all bar charts, the bars with different letters indicate significantly differences at *P* < 0.05.

## Results

### The conserved BTB-BACK and R2R3 domains are necessary for the interaction between MdBT2 and MdMYB73 proteins

**Fig. 1:**
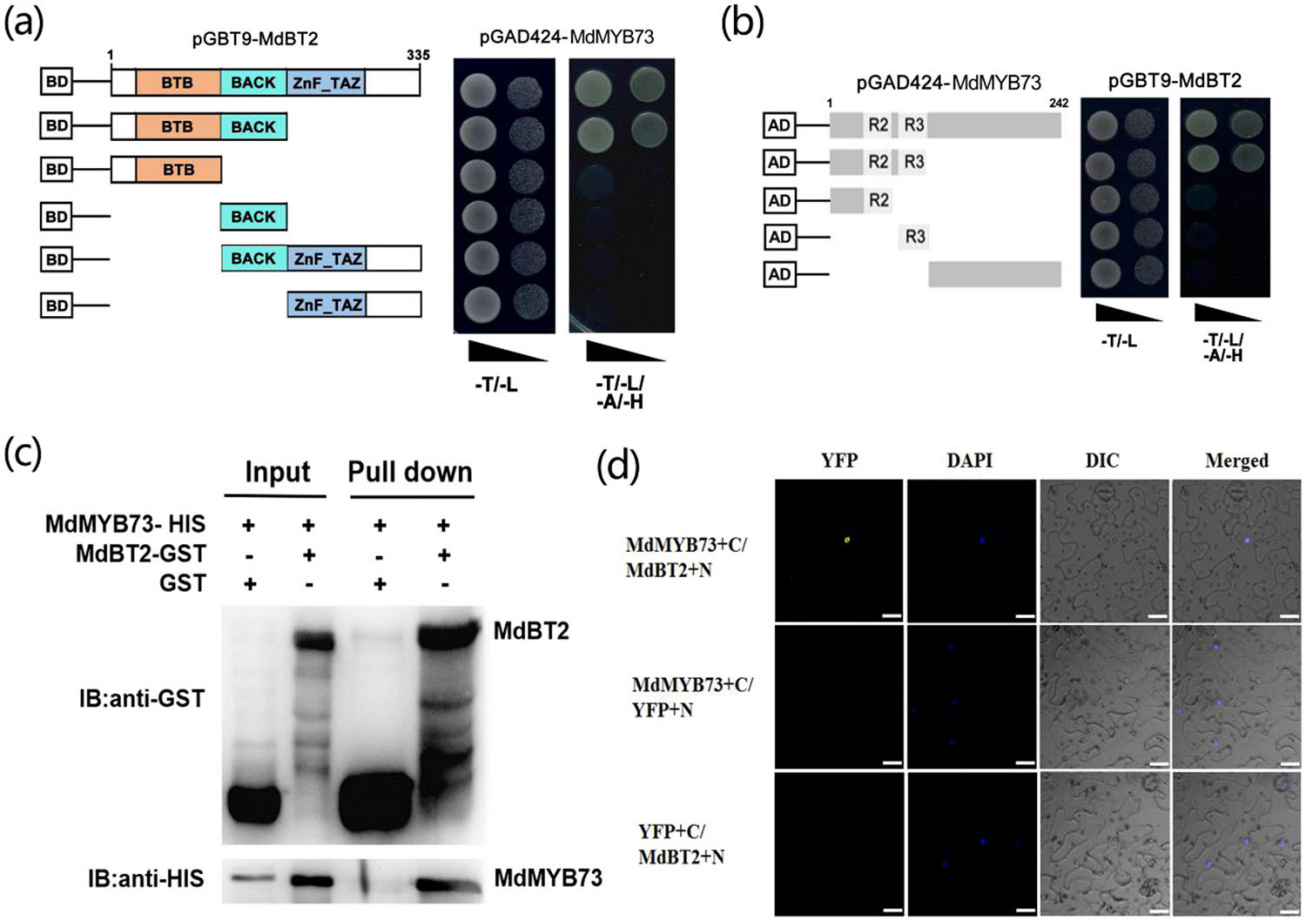
MdBT2 interacts with MdMYB73. **a**, **b** The interaction between MdBT2 and MdMYB73 in a Y2H assay. The full-length cDNA and five truncated mutants of the *MdBT2* gene (**a**) were inserted into the pGBT9 vector. The full-length cDNA of *MdMYB73* (**a**) was inserted into the pGAD424 vector. Panels show the different interactions, as indicated by yeast growth (**a**). The full-length cDNA and four truncated mutants of the *MdMYB73* gene (**b**) were inserted into the pGAD424 vector. The full-length cDNA of the *MdBT2* gene (**b**) was inserted into the pGBT9 vector. Panels show the interactions as indicated by yeast growth (**b**). **c** MdBT2 interacted with MdMYB73, as indicated by pull-down assay. MdBT2-GST expression in *E. coli* resulted in the precipitation of MdMYB73-HIS, with the anti-GST antibody and anti-HIS antibody. GST alone was used as the control. **d** MdBT2 interacted with MdMYB73 in a BiFC assay using tobacco leaf cells. MdBT2 + N and MdMYB73 + C interacted in the nucleus of tobacco leaf cells. Scale bar = 20 µm. The data were obtained from at least two biological replicates

To determine whether MdBT2 interacts with the MdMYB73 protein, we utilized Y2H assays. The MdBT2 protein contains three conserved domains, including the BTB, BACK, and ZnF_TAZ domains (Fig. 1a and Supplementary Table 2). Therefore, the full-length cDNA of the *MdBT2* gene was divided into five fragments: MdBT2^BTB-BACK^, MdBT2^BTB^, MdBT2^BACK^, MdBT2^BACK-ZnF_TAZ^, and MdBT2^ZnF_TAZ^ (Fig. 1a). Subsequently, the full-length cDNA and five truncated mutants of the *MdBT2* gene were independently inserted into the pGBT9 vector as the bait vectors, while the full-length cDNA of *MdMYB73* was inserted into the pGAD424 vector as the prey vector. The six combinations of bait and prey vectors were transformed into yeast for Y2H assays. The results indicated that both the full-length MdBT2 and truncated mutant MdBT2^BTB-BACK^ interacted with the full-length MdMYB73 protein (Fig. 1a).

To further examine the specific interaction region of MdMYB73 with the MdBT2 protein, the *MdMYB73* gene was also divided into four fragments: MdMYB73^R2R3^ (amino acids 1–131), MdMYB73^R2^ (amino acids 1–80), MdMYB73^R3^ (amino acids 81–131), and MdMYB73^None^ (amino acids 132–242). The full-length cDNA and four truncated mutants of the *MdMYB73* gene were independently inserted into the pGAD424 vector as the prey vectors. These different combinations of prey vectors with the full-length MdBT2 prey vector were transformed into yeast for use with Y2H assays. The results showed that both full-length MdMYB73 and truncated mutant MdMYB73^R2R3^ interacted with the full-length MdBT2 protein (Fig. 1b).

To further assess the interaction between MdBT2 and MdMYB73, recombinant MdBT2-GST protein and recombinant MdMYB73-HIS protein were subjected to in vitro GST pull-down assays, while the GST protein and recombinant MdMYB73-HIS protein were used as negative controls. The results showed that the GST-tagged MdBT2 protein interacted with the HIS-tagged MdMYB73 protein (Fig. 1c). In addition, bimolecular fluorescence complementation (BiFC) assays were conducted using *N. benthamiana* leaves to verify the interaction between MdBT2 and MdMYB73. MdBT2 and MdMYB73 were fused to the N-terminal and C-terminal fragments of YFP to construct the recombinant MdBT2 + N and MdMYB73 + C vectors, respectively. As a result, a nuclear-localized signal was detected in tobacco leaf cells coexpressing the MdBT2 + N and MdMYB73 + C vectors, whereas the other combinations did not show this signal (Fig. 1d).

Taken together, these results suggested that MdBT2 physically interacts with the MdMYB73 protein, and the interaction between these two proteins requires conserved BTB-BACK and R2R3 domains.

### MdBT2 affects MdMYB73 protein stability

**Fig. 2:**
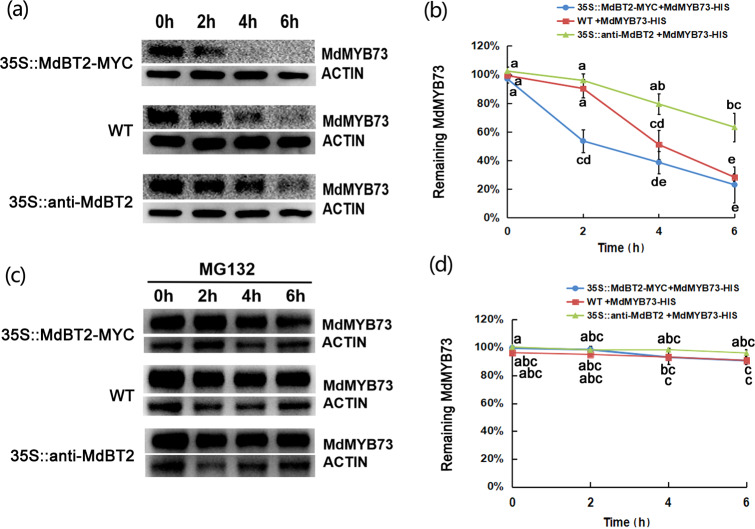
MdBT2 affects the stability of the MdMYB73 protein. **a** MdBT2 degraded MdMYB73 in vitro. Cell-free degradation assays were performed to examine the stability of the MdMYB73 protein. Three types of apple calli (WT, 35S::MdBT2, and 35S::anti-MdBT2) were coincubated with MdMYB73-HIS protein at 22 °C for the times indicated. **b** Degradation curve analysis of MdMYB73-HIS. **c** The stability of the MdMYB73 protein after MG132 treatment. WT, 35S::MdBT2, and 35S::anti-MdBT2 transgenic apple calli were treated with 50 mM MG132, and then incubated with MdMYB73-HIS at 22 °C for the times indicated. **d** Degradation curve analysis of MdMYB73-HIS upon MG132 treatment. The experiments were performed for three biological replicates, and the data are presented as the means ± standard deviation. Bars with different letters indicate significant differences at *P* < 0.05

Given that the ubiquitination-related MdBT2 scaffold protein influences the stability of its targets^24,29,36,39^, cell-free degradation assays were performed to examine the stability of the MdMYB73 protein. Three types of apple calli, WT, *MdBT2*-overexpressing apple calli (35S::MdBT2), and *MdBT2*-suppressing apple calli (35S::anti-MdBT2), were incubated with purified MdMYB73-HIS protein. As shown in Fig. 2a, b and Supplementary Fig. 1, the MdMYB73-HIS protein was degraded faster in the 35S::MdBT2 apple calli than in the WT control, while the degradation was slower in the 35S::anti-MdBT2 apple calli compared to that of the WT control.

Subsequently, the proteasome inhibitor MG132 was added to determine the MdBT2-mediated stability of the MdMYB73 protein. The cell-free degradation assays showed that MdMYB73-HIS protein abundance was unchanged in the three types of apple calli in the presence of MG132 (Fig. 2c, d and Supplementary Fig. 2).

Overall, these results suggested that MdBT2 influenced the stability of the MdMYB73 protein through the 26S proteasome pathway.

### MdBT2 facilitates the degradation of MdMYB73 protein via the ubiquitin/26S proteasome pathway

**Fig. 3:**
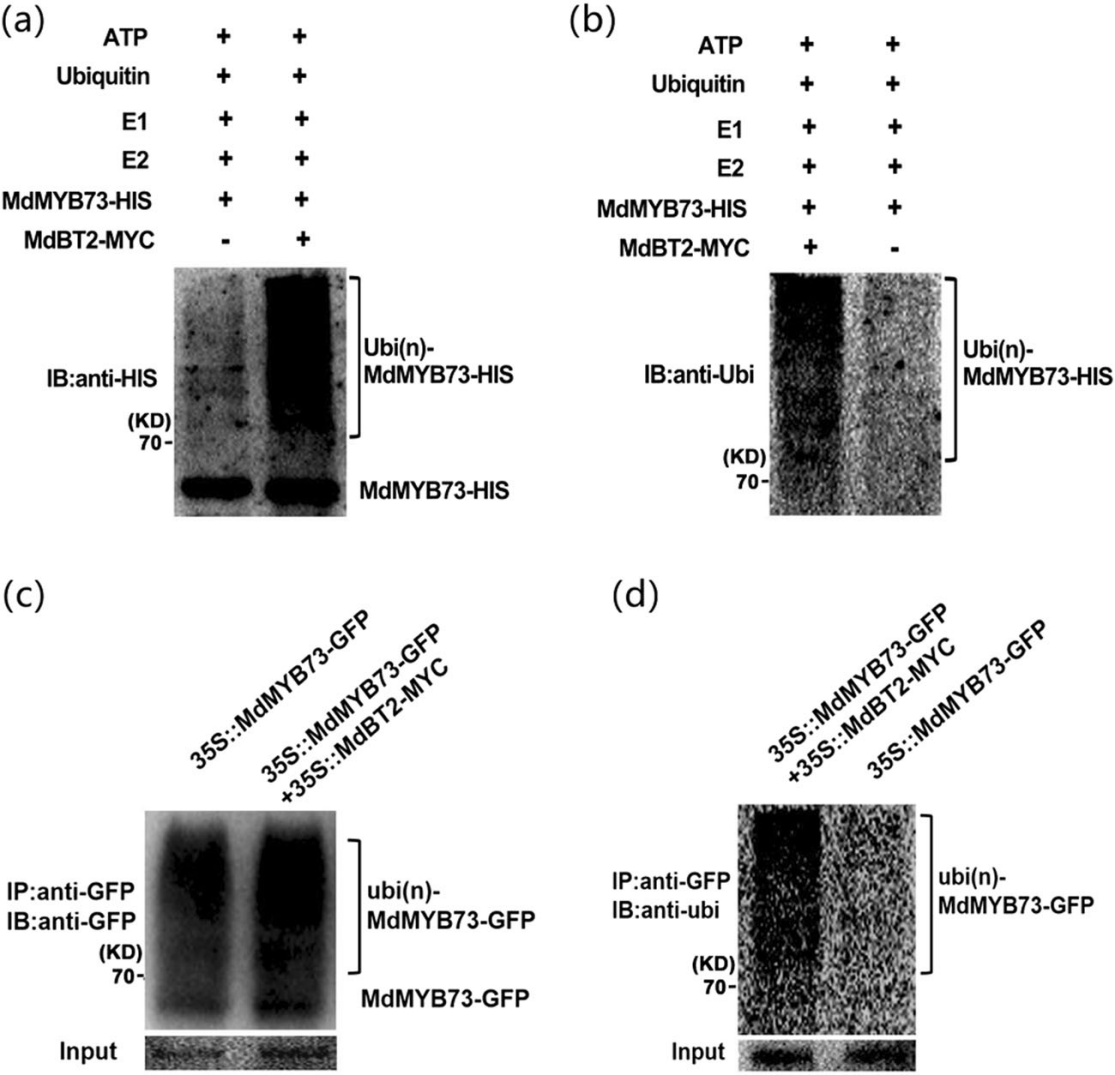
MdBT2 facilitates ubiquitination and degradation of the MdMYB73 protein. **a**, **b** Ubiquitination assays were performed in vitro. The MdBT2-MYC active protein extracted from 35S::MdBT2-MYC was incubated with MdMYB73-HIS, E1, E2, and ubi at 30 °C for immunoprecipitation, using anti-HIS (**a**) and anti-ubi (**b**) antibodies. In **a**, the left side lane had two nonspecific bands. **c**, **d** Ubiquitination assays in vivo were measured using 35S::MdMYB73-GFP and 35S::MdMYB73-GFP + 35S::MdBT2-MYC. Anti-GFP (**c**) and anti-ubi (**d**) antibodies were used to examine immunoprecipitation. The assays were performed with at least two replicates. IP Immunoprecipitated, IB immunoblotted

To confirm that MdBT2 degraded the MdMYB73 protein through the ubiquitin/26S proteasome pathway, in vitro immunoprecipitation assays were conducted using anti-HIS and anti-ubi antibodies. MdBT2-MYC active proteins extracted from 35S::MdBT2-MYC were coincubated with MdMYB73-HIS, E1, E2, and ubi in vitro for 24 h. Immunoblotting assays showed that higher amounts of high-molecular mass forms of MdMYB73 protein were detected in immunoprecipitated mixtures, using anti-HIS and anti-ubi antibodies compared with the amounts in the samples without MdBT2-MYC protein added (Fig. 3a, b).

Furthermore, in vivo ubiquitination assays were performed using two types of transgenic apple calli (35S::MdMYB73-GFP and 35S::MdMYB73-GFP + 35S::MdBT2-MYC). The immunoprecipitated protein was subsequently detected using anti-GFP and anti-ubi antibodies (Fig. 3c, d). The abundance of polyubiquitinated MdMYB73-GFP protein in 35S::MdMYB73-GFP + 35S::MdBT2-MYC apple calli was much higher than it was in the single 35S::MdMYB73-GFP apple calli. These results suggested that MdBT2 promoted the degradation of MdMYB73 protein via the ubiquitin/26S proteasome pathway.

### *MdMYB73* is required for *MdBT2* to control malate accumulation and vacuolar pH in apples

**Fig. 4:**
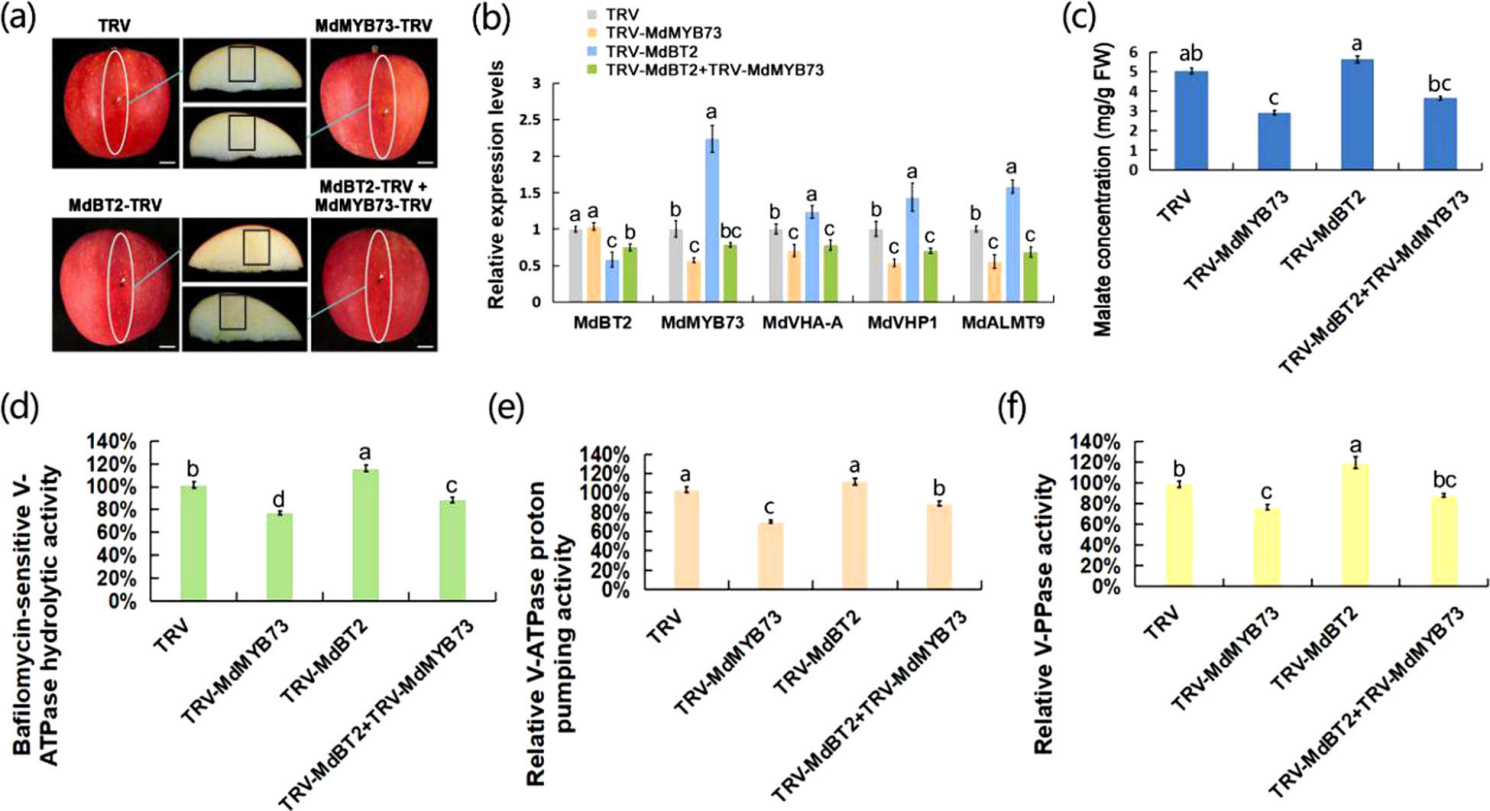
*MdMYB73* is required for *MdBT2* to control malate accumulation and vacuolar pH in apple fruit. **a** A viral vector-based method was applied to apple injection assays. TRV TRV1 + TRV2, MdBT2-TRV TRV1 + MdBT2-TRV2, MdMYB73-TRV TRV1 + MdMYB73-TRV2, MdBT2-TRV2 + MdMYB73-TRV2 TRV1 + MdBT2-TRV2 + MdMYB73-TRV2. The white arrow represents the injection point. The black box represents the sampling area. **b** qRT-PCR analysis of malate-related genes, including *MdVHA-A*, *MdVHP1*, and *MdALMT9*, in four types of viral experiments (TRV, MdBT2-TRV, MdMYB73-TRV, and MdBT2-TRV + MdMYB73-TRV). **c**–**f** Measurement of malate concentration (**c**), hydrolytic (**d**), and proton-pumping activity levels (**e**) of V-ATPase and V-PPase (**f**) in four types of viral experiments (TRV, MdBT2-TRV, MdMYB73-TRV, and MdBT2-TRV + MdMYB73-TRV). Bars with different letters are significantly different at *P* < 0.05, according to Tukey’s single factor tests. Data are shown as the means ± SD

To characterize whether and how MdBT2 controls malate accumulation, a viral vector-based method was applied by injection into apple fruits. A blue dye was initially injected into the apple fruits to observe the injection pathway (Supplementary Fig. 3). Subsequently, four types of viral constructs (TRV, TRV-MdBT2, TRV-MdMYB73, and TRV-MdBT2 + TRV-MdMYB73) were obtained for fruit infiltration assays (Fig. 4a). The qRT-PCR assays showed that the relative transcript levels of the *MdBT2* gene were 0.58 and 0.75 after being separately infiltrated with TRV-MdBT2 and TRV-MdBT2 + TRV-MdMYB73, respectively, compared with TRV. The relative transcript levels of the *MdMYB73* gene were 0.57 and 0.78 after being separately infiltrated with TRV-MdMYB73 and TRV-MdBT2 + TRV-MdMYB73, respectively, compared with TRV (Fig. 4b). These results indicated that the infiltration assays were successful. As the direct downstream target genes of the MdMYB73 TF, the transcript levels of *MdVHA-A*, *MdVHP1*, and *MdALMT9* decreased with the decrease in *MdMYB73* gene expression. The transcript levels of *MdMYB73* and its direct downstream genes were negatively associated with the *MdBT2* gene transcript level (Fig. 4b). Subsequently, the hydrolytic and proton-pumping activity levels of V-ATPase and V-PPase were determined. V-ATPase and V-PPase activity levels in the TRV-MdMYB73-injected areas were much lower than those in the TRV control. However, TRV-MdBT2-injected areas had much higher V-ATPase and V-PPase activities than those of the TRV control (Fig. 4d–f). Furthermore, TRV-MdMYB73 counteracted the effect of TRV-MdBT2 in TRV-MdBT2 + TRV-MdMYB73-injected areas (Fig. 4d–f), suggesting that MdBT2 influenced V-ATPase and V-PPase activities, depending on the presence of MdMYB73. As expected, the malate concentration was in agreement with the V-ATPase and V-PPase activity levels; the malate content was reduced in the TRV-MdMYB73 and TRV-MdBT2 + TRV-MdMYB73-injected areas compared to that of the control (ab), and only MdMYB73-TRV (c) showed reduced malate concentration. MdBT2-TRV (a) and MdBT2-TRV + MdMYB73-TRV (bc) were not significantly different from the control (Fig. 4c).

**Fig. 5:**
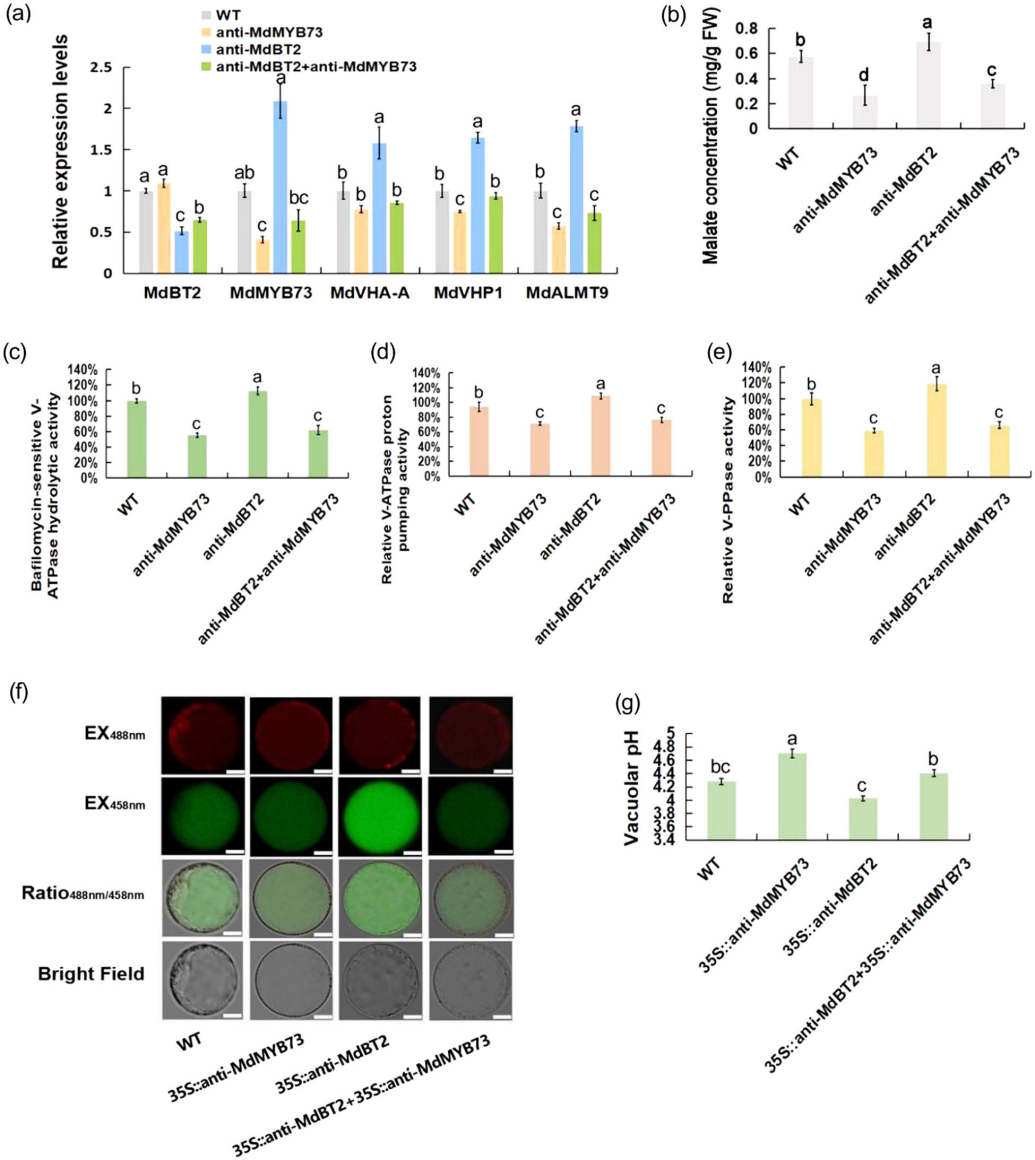
*MdMYB73* is required for *MdBT2* to control malate accumulation and vacuolar pH in apple calli. **a** Expression analysis of malate-related genes, *MdVHA-A*, *MdVHP1*, and *MdALMT9*, using qRT-PCR in WT and transgenic apple calli (35S::anti-MdMYB73, 35S::anti-MdBT2, and 35S::anti-MdBT2 + 35S::anti-MdMYB73). **b** The malate concentration in the WT and transgenic apple calli (35S::anti-MdMYB73, 35S::anti-MdBT2, and 35S::anti-MdBT2 + 35S::anti-MdMYB73). **c**–**e** Hydrolytic (**c**) and proton-pumping activity levels of V-ATPase (**d**) and V-PPase (**e**) in WT and transgenic apple calli (35S::anti-MdMYB73, 35S::anti-MdBT2, and 35S::anti-MdBT2 + 35S::anti-MdMYB73). **f**, **g** Emission intensities (**f**) and quantitative analysis (**g**) of protoplasts to determine the vacuolar pH in apple calli with BCECF dyes at 488 nm and 458 nm (WT, 35S::anti-MdBT2, 35S::anti-MdMYB73, and 35S::anti-MdBT2 + 35S::anti-MdMYB73). Scale bar = 10 µm. Bars with different letters are significantly different at *P* < 0.05, according to Tukey’s single factor tests. Data are shown as the means ± SD

Four types of apple calli (WT, 35S::anti-MdBT2, 35S::anti-MdMYB73, and 35S::anti-MdBT2 + 35S::anti-MdMYB73) were used to study the regulation of malate accumulation. The *MdBT2* and *MdMYB73* genes were successfully suppressed in the corresponding calli compared to the WT control (Fig. 5a), indicating that the genetic transformation in apple calli was successful. As downstream genes of MdMYB73, the *MdVHA-A*, *MdVHP1*, and *MdALMT9* genes exhibited reduced transcript levels in the 35S::anti-MdMYB73 and 35S::anti-MdBT2 + 35S::anti-MdMYB73 samples, and they were increased in the 35S::anti-MdBT2 sample (Fig. 5a).

Next, malate concentration and the hydrolytic and proton-pumping activity levels of V-ATPase and V-PPase were also examined. This analysis revealed that malate concentration V-ATPase and V-PPase activity levels in the 35S::anti-MdMYB73 apple calli were much lower than those in the WT control. However, 35S::anti-MdBT2 calli had a much higher malate concentration and V-ATPase and V-PPase activity levels compared with those of the WT control (Fig. 5b–e). Moreover, 35S::anti-MdMYB73 counteracted the effect of 35S::anti-MdBT2 in the 35S::anti-MdBT2 + 35S::anti-MdMYB73 calli (Fig. 5b–e), suggesting that MdBT2 influenced malate concentration, as well as V-ATPase and V-PPase activity levels, depending on the presence of MdMYB73. In addition, the vacuolar pH in the apple calli was measured using a BCECF-AM ratiometric fluorescent pH indicator. As shown in Fig. 5f, g, the average vacuolar pH in the WT, 35S::anti-MdMYB73, and 35S::anti-MdBT2 calli was 4.28, 4.70, and 4.03, respectively, while the vacuolar pH in the 35S::anti-MdBT2 + 35S::anti-MdMYB73 calli was 4.41. These results indicated that only the silencing of *MdMYB73* increased vacuolar pH, while the silencing of *MdBT2* or *MdBT2*, and *MdMYB73* genes did not change vacuolar pH compared to that of the control.

These results support our hypothesis that MdMYB73 is required for MdBT2 to control malate accumulation and vacuolar pH.

### MdBT2 negatively regulates the *MdMYB73*-downstream gene *MdALMT9*

**Fig. 6:**
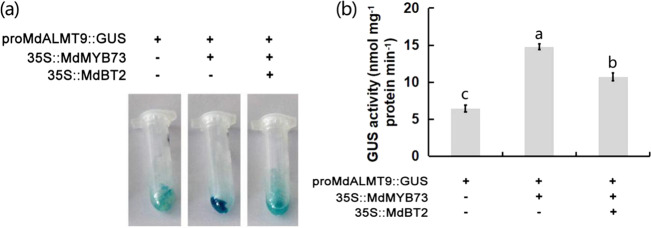
MdBT2 negatively regulates the *MdMYB73*-downstream gene *MdALMT9*. **a** GUS assays in different apple calli (35S::MdBT2 + 35 S::MdMYB73 + proMdALMT9::GUS, proMdALMT9::GUS + 35S::MdMYB73, and proMdALMT9::GUS). **b** GUS activity measured using a fluorescence spectrometer with excitation at 365 nm and emission at 455 nm in different apple calli (35S::MdBT2 + 35 S::MdMYB73 + proMdALMT9::GUS, proMdALMT9::GUS + 35S::MdMYB73, and proMdALMT9::GUS). Bars with different letters are significantly different at *P* < 0.05, according to Tukey’s single factor tests. Data are shown as the means ± SD

GUS assays were performed to verify the effect of MdBT2 on the transcriptional activity of *MdALMT9* using proMdALMT9::GUS, with proMdALMT9::GUS + 35S::MdMYB73 used to detect GUS enzyme activity. This analysis revealed that proMdALMT9::GUS + 35S::MdMYB73 generated higher GUS enzyme activity than proMdALMT9::GUS alone (Fig. 6a, b). However, GUS enzyme activity decreased when 35S::MdBT2 was transformed into proMdALMT9::GUS + 35S::MdMYB73, suggesting that MdBT2 negatively regulated *MdALMT9* transcriptional activity via the MdBT2-MdMYB73-MdALMT9 pathway.

## Discussion

Malate is a pivotal metabolite that is involved in regulating osmotic pressure, pH homeostasis, and stress resistance, and it significantly influences fruit quality^2,7^. A previous study has shown that the BTB-BACK-TAZ protein MdBT2 ubiquitinates the bHLH TF MdCIbHLH1 to regulate malate accumulation and vacuolar acidification^24^. In this study, we discovered that malate accumulation and vacuolar acidification are also regulated, by MdBT2 ubiquitinating the MYB TF MdMYB73 in apple.

Acting as an important posttranslational modification, ubiquitination plays an important role in regulating protein activity in eukaryotes, with significant impacts on plant growth and development^34,46–48^. There are some studies on the transcriptional regulation of malate accumulation, but posttranslational regulatory mechanisms, such as ubiquitination have not been studied extensively^2,5,12,24^. In addition to the previously identified MdCIbHLH1 TF, we found that an MdMYB73 TF was ubiquitinated by MdBT2 to regulate malate accumulation and vacuolar acidification. MdCIbHLH1 and MdMYB73 both function as TFs and are targets of MdBT2. Remarkably, MdCIbHLH1 also interacted with MdMYB73 to form the MBW complex, which enhanced the effect on downstream target genes to modulate malate accumulation and vacuolar pH^2^.

As a scaffold protein, BT2 recruits E3 ubiquitin ligases, such as CUL3 and RBX1 to form a complex for the ubiquitination and degradation of target proteins^26,27^. Recently, MdBT2 was found to be involved in ubiquitination to regulate iron homeostasis depending on the ubiquitin ligase MdCUL3 (ref. ^39^). MdBT2 facilitated the ubiquitination of MdMYB1 to regulate the anthocyanin production through an MdCUL3-independent pathway^36^. Interestingly, the TAZ domain of MdBT2 was required for the interaction between MdBT2 and MdbHLH104, which regulated iron homeostasis, while the BACK domain of MdBT2 was required for the MdBT2-MdMYB1 protein interaction^36,39^. In the current study, we found that the conserved BTB-BACK domain was necessary for the interaction between MdBT2 and MdMYB73 proteins (Fig. 1). Therefore, we speculate that MdBT2 can accelerate the ubiquitination of MdMYB73, possibly through an MdCUL3-independent pathway. However, the determination of whether MdBT2 ubiquitinates MdMYB73 by recruiting a ubiquitin E3 ligase (RBX1 or others) remains for further examination.

MdMYB73 promotes malate accumulation by activating proton pumps and secondary transporters, such as MdALMT9, MdVHA-A, and MdVHP1 (ref. ^2^). In addition, another MYB TF (MdMYB1) plays a central role in malate accumulation by targeting MdVHA-Bs, MdVHA-E, MdVHP1, and MdtDT^12^. Although MdMYB73 and MdMYB1 have similar functions in regulating malate accumulation, the downstream target genes of MdMYB1 and MdMYB73 differ. These findings clearly imply that MYB TFs play important roles in malate accumulation, but by different pathways. Interestingly, MdBT2 interacts with both MdMYB1 and MdMYB73 (Fig. 1)^36^. MdMYB1 regulates both anthocyanin and malate accumulation, and MdBT2 regulates the accumulation of MdMYB1-mediated anthocyanin^12,36^. However, whether MdBT2 affects malate accumulation through MdMYB1 still needs further study. The role of MdALMT9 in regulating malate accumulation has been clearly demonstrated^2,5,7,20^, and MdMYB73 regulates malate accumulation by activating the transcription of MdALMT9 (ref. ^2^). In this study, the effect of MdBT2 on malate accumulation through MdMYB73-MdALMT9 was confirmed by the lower GUS enzyme activity seen in 35S::MdBT2 + 35S::MdMYB73 + proMdALMT9::GUS (Fig. 6). Compared to the anthocyanin accumulation regulated by MdBT2-MdMYB1 (ref. ^36^), malate accumulation was regulated by MdBT2-MdMYB73. It is currently unknown whether MdMYB73 affects anthocyanin accumulation or whether MdBT2 also regulates MdMYB73-mediated anthocyanin accumulation; therefore, further studies are needed.

The BTB-BACK-TAZ protein, MdBT2, plays various roles in the integration of signals involving light, temperature, carbon and nitrogen nutrition, hormones, and stresses in plants^28,29,36,39^. In apple, the ABA-responsive protein MdBT2 interacts directly with MdbHLH93 and degrades the MdbHLH93 protein by the ubiquitin/26S proteasome pathway, resulting in delayed leaf senescence^49^. In addition, MdBT2 is also a nitrate-responsive protein that regulates anthocyanin biosynthesis by interacting with the MdMYB1 TF in apple^36^. MdBT2 also regulates plant cold tolerance by promoting the degradation of the MdMYB23 protein^29^. Previous research indicating that MdBT2 affects malate accumulation by targeting MdMYB73 was also verified in this study. Thus, we propose that different external environmental factors, such as nitrate signaling, light or cold, can regulate malate accumulation.

**Fig. 7:**
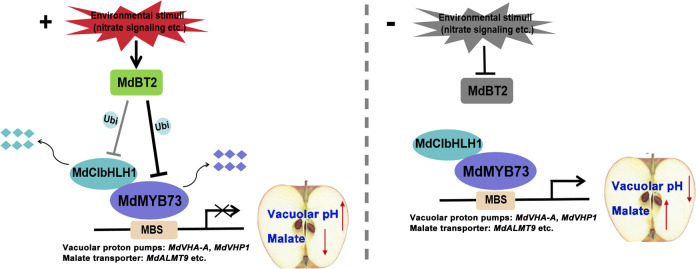
Working model showing that the BTB-BACK-TAZ domain protein MdBT2 modulates malate accumulation and vacuolar pH. Left, with environmental stimuli (such as nitrate signaling), MdBT2 ubiquitinates MdCIbHLH1 and MdMYB73 for degradation, which reduces the transcription of malate-associated genes by MdMYB73, leading to lower malate accumulation and higher vacuolar pH. Right, with no environmental stimulus, MdCIbHLH1 enhances the activity of MdMYB73, promoting the transcription of malate-associated genes, thus resulting in higher malate accumulation and lower vacuolar pH

Taken together, our discoveries and those of previous studies can be integrated into a working model (Fig. 7). This working model depicts a mechanism, in which the BTB-BACK-TAZ protein MdBT2 regulates malate accumulation and vacuolar pH. With environmental stimuli (such as nitrate signaling), MdBT2 ubiquitinates MdCIbHLH1 and MdMYB73 to target them for degradation, which reduces the transcription of malate-associated genes by MdMYB73, leading to lower malate accumulation and high vacuolar pH. When there are no environmental stimulus, MdCIbHLH1 enhances the activity of MdMYB73, promoting the transcription of malate-associated genes, thus resulting in high malate accumulation and lower vacuolar pH. Overall, our findings provide new insights into the molecular mechanism and regulatory pathway of the ubiquitination of relevant TFs, affecting malate accumulation and expand the current understanding of malate regulation mechanisms. This information may be of particular significance in guiding traditional breeding programs and fruit tree molecular breeding, as well as improving fruit quality and stress tolerance.

## Supplementary information

Supporting Information
