# Supplementary material for: BTB-BACK-TAZ domain protein MdBT2-mediated MdMYB73 ubiquitination negatively regulates malate accumulation and vacuolar acidification in apple
Source: Hortic Res. 2020 Sep 2;7:151. doi: 10.1038/s41438-020-00384-z (PMC7468283; doi:10.1038/s41438-020-00384-z)
Supplement: Supplementary file 1 — Supporting Information [file 41438_2020_384_MOESM1_ESM.docx]

**
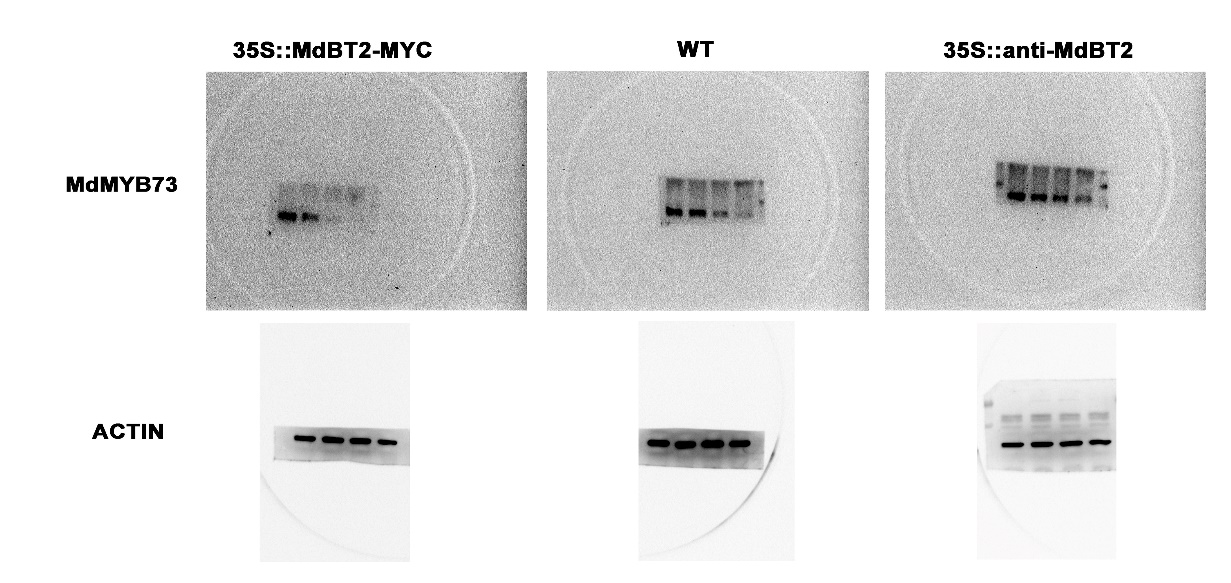
**

**Supplementary Fig. 1 The stability of MdMYB73 protein.**

**
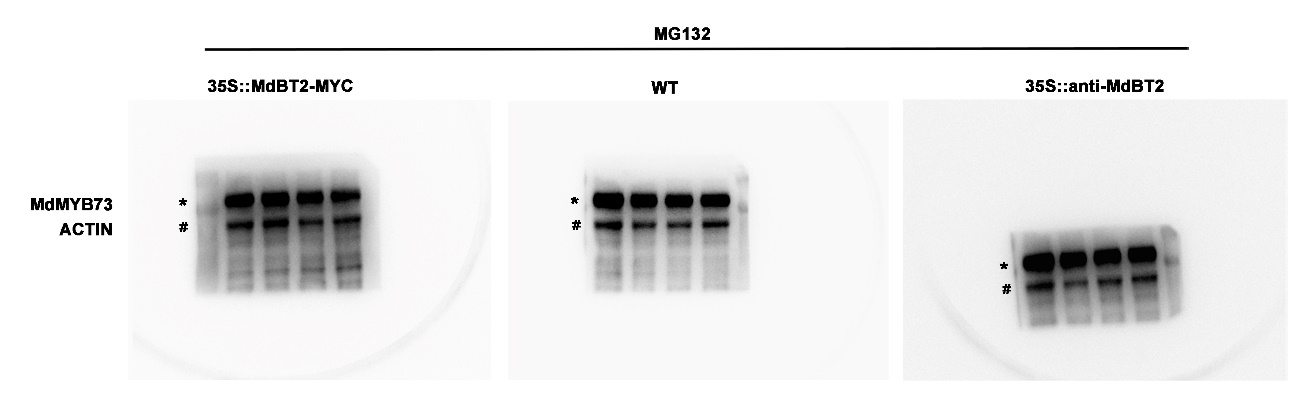
**

**Supplementary Fig. 2 The stability of MdMYB73 protein under MG132 treatment.** Note: the asterisk and hash sign represent MdMYB73 and ACTIN proteins, respectively.

**
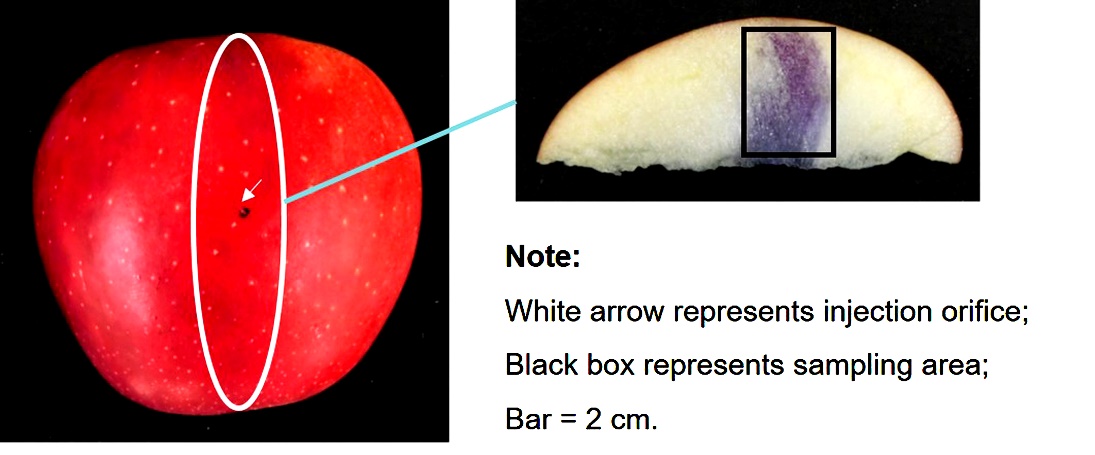
**

**Supplementary Fig. 3** Staining experiments of dye-injected apple.

**Supplementary Table 1** The primers used in this study.

| **Name** | **Primer sequences** |
| --- | --- |
| MdMYB73-F(RT-qPCR) | AGTAGAGAGCAGATATCCGAC |
| MdMYB73-R(RT-qPCR) | ATCATGATCATGCACTGCTAC |
| MdVHA-A-F(RT-qPCR) | TGGAGAACGTGGAAATGAAATG |
| MdVHA-A-R(RT-qPCR) | CTTCACGACCATCAGGCAAA |
| MdVHP1-F(RT-qPCR) | CTGCAGCACGGTCAAATTA |
| MdVHP1-R(RT-qPCR) | CGCGCACACACACATATACA |
| MdALMT9-F(RT-qPCR) | TCTTGTTTTGTAATTAACT |
| MdALMT9-R(RT-qPCR) | GAGGAAAGAAAAGTTAATT |
| proMdALMT9::GUS-F | ACGTCCCAGAGTTTCTTGTTC |
| proMdALMT9::GUS-R | GTTGGAAGAGGGAAAGTGG |
| MdBT2-F(RT-qPCR) | CTGGCGAAGATGTGCGAT |
| MdBT2-R(RT-qPCR) | CTCTCTATGCCTCCTTGTCCTC |
| TRV-MdMYB73-F | GGATCCATTTAATCTATGAAGCTCC |
| TRV-MdMYB73-R | CTCGAGAGTAATGAGGGGATGAGCA |
| TRV-MdBT2-F | GAATTCTGCAGCAAGAGAAGAAGAC |
| TRV-MdBT2-R | TCTAGACGACTAATTAATCACAATCTG |
| BD-MdBT2-F | GAATTCATGGAAGCTAATCCGACCGC |
| BD-MdBT2-R | CTGCAGCGACTAATTAATCACAATCTG |
| AD-MdMYB73-F | GAATTCATGGAAGCGATGAATATGTGC |
| AD-MdMYB73-R | GGATCCAATTTAATCTATGAAGCTCCG |
| AD-MdMYB73-F(1-240) | GAATTCATGGAAGCGATGAATATGTGC |
| AD-MdMYB73-R(1-240) | GGATCCAAGCTGGTTGCACCACCTGAG |
| AD-MdMYB73-F(1-393) | GAATTCATGGAAGCGATGAATATGTGC |
| AD-MdMYB73-R(1-393) | GGATCCCAACGTCGAGTTCCAGTGAT |
| AD-MdMYB73-F(241-393) | GAATTCAGCCCCAGCGTCCAGCATCG |
| AD-MdMYB73-R(241-393) | GGATCCCAACGTCGAGTTCCAGTGAT |
| AD-MdMYB73-F(394-726) | GAATTCAAGCGGAGGGTCAGGGGAGA |
| AD-MdMYB73-R(394-726) | GGATCCATTTAATCTATGAAGCTCCG |

**Supplementary Table 2** The amino acids sequences of MdBT2.

MEANPTATSGTSVDLYGLSGTKYLPEPDVDILTCDAIRIPVHSCILASVSPVLENIIDRPRKHRSSERVIPILGVPYDAVLAFVRFLYSSRCTEENMEKYGIHLLALSHVYLVPQLKNRCTKELGQRLTIENVVDVLQLAKMCDAADLYLKCMKLVANHFKVVETTEGWKFLQAHDPWLELHIMQFIDEIESRKKRTRRHREEQRLYLQLSEAMECLEHICKEGCTSVGPYDMEPGYKKGPCSKFSTCQGLQMLIQHFATCKRRVNGGCLRCKRMWQLLKLHSSMCEEPDSCRVPLCRQFKLKMQQEKKTDDARWKLLVKKVMSAKTLSSLSLPKRKREEELGEGRSTSTITAHGIRSFRL

Note: these font colors mean the BTB domain, BACK domain, and the ZnF_TAZ domain, respectively.
